# Supportive supervision of close-to-community providers of health care: Findings from action research conducted in two counties in Kenya

**DOI:** 10.1371/journal.pone.0216444

**Published:** 2019-05-29

**Authors:** Robinson Njoroge Karuga, Maryline Mireku, Nelly Muturi, Rosalind McCollum, Frederique Vallieres, Meghan Kumar, Miriam Taegtmeyer, Lilian Otiso

**Affiliations:** 1 LVCT Health, Department of Research and Strategic Information, Nairobi, Kenya; 2 Liverpool School of Tropical Medicine, Department of International Public Health, Liverpool, United Kingdom; 3 Centre for Global Health, School of Psychology, Trinity College, Dublin, Ireland; Public Library of Science, UNITED KINGDOM

## Abstract

**Background:**

Close-to-community (CTC) providers of health care are a crucial workforce for delivery of high-quality and universal health coverage. There is limited evidence on the effect of training supervisors of this cadre in supportive supervision. Our study aimed to demonstrate the effects of a training intervention on the approach to and frequency of supervision of CTC providers of health care.

**Methods:**

We conducted a context analysis in 2013 in two Kenyan counties to assess factors that influenced delivery of community health services. Supervision was identified a priority factor that needed to be addressed to improve community health services. Supervision was inadequate due to lack of supervisor capacity in supportive approaches and lack of supervision guidelines. We designed a six-day training intervention and trained 48 purposively selected CTC supervisors on the educative, administrative and supportive components of supportive supervision, problem solving and advocacy and provided them with checklists to guide supervision sessions. We administered quantitative questionnaires to supervisors to assess changes in supervision frequency before and after the training and then explored perspectives on the intervention with community health volunteers (CHVs) and their supervisors using qualitative in-depth interviews.

**Results:**

Six months after the intervention, we observed that supervisors had shifted the supervision approach from being controlling and administrative to coaching, mentorship and problem solving. Changes in the frequency of supervision were found in Kitui only, whereby significant decreases in group supervision were met with increases in accompanied home visit supervision. Supervisors and CHVs reported the intervention was helpful and it responded to capacity gaps in supervision of CHVs.

**Conclusion:**

Our intervention responded to capacity gaps in supervision and contributed to enhanced supervision capacity among supervisors. Supervisors found the curriculum acceptable and useful in improving supervision skills.

## Background

Close-to-community (CTC) providers of health care in low- and middle-income countries (LMICs) have played a key role in increasing equitable access to preventive and basic curative services and achieving universal health coverage (UHC). This is particularly important for marginalized and poor communities [1]. CTC providers of health care include lay community members who (mainly) volunteer their services and formal primary health care workers who provide technical support and supervision to these lay health providers [2, 3]. Since they are uniquely embedded in community settings, CTC providers play a critical role in community-level disease surveillance, health education, mobilization for crucial public health interventions (e.g. maternal and newborn care; water, sanitation and hygiene (WASH); family planning, among others) [4]. There is global consensus that investing in CTC health programs is a vital strategy for achieving health-related sustainable development goals by extending healthcare to disadvantaged populations, hence promoting equity in health [5]. Scaling-up of existing CTC health programs has the potential to save up to three million lives annually and can yield returns on investments of up to 1000% due to increased productivity and employment in healthier communities [6].

In recognition of the importance of community health and in response to worsening maternal, newborn and child health indicators, Kenya’s Ministry of Health launched the national Community Health Strategy in 2006 [7]. This strategy aimed to expand community access to healthcare across all stages of the human life cycle. Kenya’s community health strategy places community units at the center of the community health approach. A community unit is a geographical area composed of an average of 5,000 people [7]. Each community unit should be served by two categories of CTC providers of health care in Kenya, namely: a) Community Health Extension Workers (CHEWs)–formal public health sector employees linking communities with the formal health system; and b) Community Health Volunteers (CHVs)—community members who volunteer to provide health promotion, health education, basic curative and referral services [7]. CHEWs have supervisory responsibilities over CHVs. Since 2013, the responsibility of delivering and managing community health services was devolved to 47 county governments after promulgation of Kenya’s new constitution, while the national level Ministry of Health is responsible for policy formulation, development of guidelines and providing technical assistance to county departments of health.

Similar to other LMICs, Kenya faces systemic challenges in the provision of community health services that affect performance of CTC providers of health care. These challenges include high attrition of CTC providers of health care; lack of supplies and logistical support; low morale among CTC providers of health care; and inadequate supervision [8–12]. Inadequate supervision of CTC providers of health care is linked to poor performance. According to published literature, CTC providers of health care are not adequately supervised due to insufficient training of supervisors in supervision [9, 13–15]; heavy administrative workloads of supervisors; lack of supportive supervision guidelines; and insufficient operational support to provide supportive supervision [16–20]. Supportive supervision promotes quality at all levels of the health system by strengthening relationships within the system, focusing on problem identification, problem resolution and helping health workers to optimize the allocation of resources [21].

Supportive supervision of CTC providers of health care contributes to improvement in their performance and retention in community health services [10, 22, 23]. This supervision approach involves dialogue between supervisors and supervisees to establish clear goals and identify solution to problems, emphasizing the inter-personal nature of supervision, through joint problem solving and action planning [19, 21]. When done consistently, supportive supervision can provide a mechanism for professional development, thereby increasing motivation and performance of CTC providers of healthcare [10, 11, 13, 16, 24]. There are several approaches that can qualify as supportive supervision, such as: group supervision (a group of CTC providers of health care meet with a supervisor to discuss community health data, challenges and continuing education); one-on-one supervision (supervisors meet one CTC provider of health care at a time for individual support and discussion of performance); accompanied home visits (supervisors accompany CTC providers of health care during household visits and provide support and guidance). There is little evidence on the effect of training interventions that aim to enhance supportive supervision of CTC providers of health care in LMICs.

**Fig 1:**
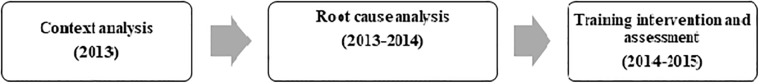
Flow of study activities in this action research.

This paper presents findings of an action research study that sought to establish supportive supervision in two counties in Kenya. Using the action research approach, we first conducted a context analysis to assess factors that influence the delivery of CTC health services and performance of CTC providers of health care in Kenya. Using findings from the context analysis, we conducted a root cause analysis with stakeholders to prioritize which factors to address with an intervention and selected supervision. Again based on the context analysis findings, we designed a training intervention to improve supervision of CTC health providers. We implemented and assessed this intervention between 2014 and 2015, as illustrated in Fig 1. Our primary outcome for enhancing supervision was the incorporation of facilitative approaches in supervision as described by Hill *et al*., (2014) [13], and our secondary outcome was to assess the effect of the intervention on frequency of supervision. Supervisors and CTC providers were participants in both the research and the process of implementing the intervention [25].

This research was part of a five-year multi-country program called REACHOUT. The REACHOUT program aimed to maximize the equity, effectiveness and efficiency of CTC health services in rural areas and urban slums in six countries: Bangladesh, Ethiopia, Indonesia, Kenya, Malawi and Mozambique [26].

## Methods

In this section, we first summarize how we conducted the context analysis to assess factors that influence delivery of community health services and how we designed an intervention to establish supportive supervision in the two study counties. We then describe how we implemented and assessed the intervention.

### Study sites

We purposively selected Nairobi and Kitui as our study counties, out of the 47 counties, to represent a rural and urban-slum setting, respectively. Kitui County is semi-arid and is located towards the south-east of Kenya, while Nairobi County is the capital city of Kenya whose population covers a diverse urban socio-demographic spectrum, with vast economic disparities [27]. In each of these counties, we purposively selected two community units in consultation with the county administrators responsible for community health services.

### Context analysis

In the context analysis, we took an exploratory qualitative approach to assess factors that influenced delivery of community health services in Kenya. This involved reviewing peer-reviewed and grey literature on community health services; mapping of CTC providers of health care; and collection of primary qualitative data from policy makers, health managers and CTC providers of health care. Three researchers reviewed literature on community health services in Kenya by gathering secondary data from both international and local health-oriented online sources such as PLOS One, Medline, Popline, PubMed and Science Direct. Examples of search terms that we used in the online literature searches were ‘community health worker’, ‘community health work’, ‘community health strategy’, ‘volunteer health worker’, and ‘lay health worker’. In addition, we reviewed policy documents and project reports related to community health services in Kenya that we obtained from the Ministry of Health (MoH) and organizations working with CTC providers of health care. Additionally, a team of five researchers mapped all stakeholders involved in community health services. Findings of the stakeholder mapping exercise were published in detail by Mireku *et al*., [18].

In the context analysis, we conducted focus group discussions (FGDs) with 72 (23 male, 49 female purposively sampled CHVs to explore their perceptions on how supervision was carried out and the factors that enabled and limited supervision. We sampled these CHVs with support from CHEWs in the purposively selected community units because they had direct access to these CHVs. Trained field assistants and researchers moderated these FGDs using pre-tested discussion guides. On average, FGDs lasted about one and a half hours. Using in-depth interviews (IDIs), we explored policy makers’ and health system managers’ perceptions of how human resource planning and management affected the performance of CTC health providers of health care. We purposively selected participants based on their knowledge and involvement in the community health strategy, as either implementers or decision makers. For IDIs, we recruited four (3 male, 1 female) policy makers in the national MoH, seven (3 male, 4 female) sub-county level health managers and 16 CHEWs (9 male, 7 female) for IDIs that lasted an average of 50 minutes. We piloted the topic guides before embarking on fieldwork with the data collection team after a three-day training on the study protocol, data collection techniques. A team of qualitative researchers inductively developed the coding framework based on the content of the FGD and IDI transcripts during an analysis workshop. Joint open coding was done by the team to arrive at a consensus on the themes in the coding framework. To enhance trustworthiness of the analysis, double coding was done with selected transcripts in Nvivo 10 [28]. A point of saturation was reached when no new themes emerged from the qualitative data in the transcripts. The key finding in the context analysis was that supervision of CTC providers of health care providers was inadequate and unsupportive. Comprehensive methods (data collection, sampling, analysis) and findings of the context analysis have been published by Mireku *et al*. (2014) and are available at this link http://www.reachoutconsortium.org/media/1837/kenyacontextanalysisjul2014compressed.pdf [18].

### Root cause analysis

**Fig 2:**
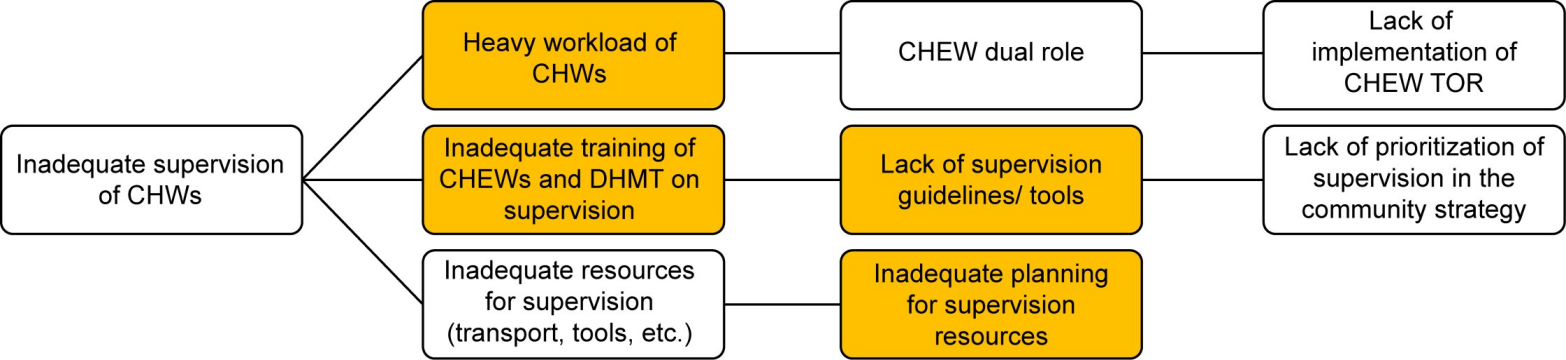
Root-cause analysis used to determine the factors that led to inadequate supervision of CTC health providers [17, 18].

Following the context analysis, the research team convened an analysis workshop in November 2013 to determine key factors and root causes that contributed to inadequate supervision of CTC providers of health. We applied the problem tree approach to identify the root causes of inadequate supervision as illustrated in Fig 2 [17, 18]. We then presented our root cause analyses to community health stakeholders for their input. Our stakeholders comprised members of a national level technical working group for community health services, sub-county health management teams and CTC providers of health care in the study sites. All stakeholder meetings used the priority matrix to reflect on each of the factors identified in the context analysis and ranked them based on priority. Using the priority matrix, stakeholders ranked inadequate supervision as the factor that required most urgent intervention. This processes informed the design of the intervention.

### Intervention to improve supportive supervision

Between August 2014 and July 2015, we designed, implemented and assessed a training intervention that aimed to improve supervision of CTC providers of health care in the same sites where we conducted the context analysis. We applied the action research approach in this phase of the project. A team of curriculum developers and researchers from LVCT Health and the Liverpool School of Tropical Medicine, in consultation with Kenya’s national MoH, developed a six-day training manual for supportive supervision of CTC providers of health care. This training manual was adapted from the Kenya’s National Training Manual for Supportive Supervision used for training home-based HIV testing and counselling (HBTC) supervisors. At the time of this study, this manual was only being utilized by non-governmental organizations to train supervisors of CTC health care providers involved in HBTC at community level. Contents of the training manual covered topics on (i) ***supportive roles*** (workers’ welfare); (ii) ***administrative roles*** (performance related issues); and (iii) ***educative roles*** (capacity building). Additional modules on problem solving skills, advocacy, and identification of problems, prioritization and developing action plans were incorporated into the training manual. We then developed supervision checklists to guide supervisors on how to prepare for supervision sessions and how to carry out the supportive, administrative and educative functions of supportive supervision. These checklists are available as supplementary information S1–S4 Files.

We piloted the training manual and supervision checklists among CHEWs and members of a sub-county Health Management Team in a non-research community unit within Nairobi County. We revised the contents of the training manual and training approaches based on lessons learned and feedback from this pilot. Between May and July 2015, trainers from the LVCT Health Training Institute delivered the six-day training in workshops to CHEWs, sub-county health managers in charge of community health services and peer supervisors of CHVs in both study counties. After the training, we provided participants with supervision checklists and gave them six months to implement skills they had learned during the workshops. The original plan was to evaluate the intervention after 12 months of implementation. However, administrative and political changes after the 2013 general elections delayed implementation of the study. Specifically, in 2013, health services were devolved to 47 newly formed counties, which disrupted delivery of health care delivery through reorganization of the health workforce and decision-making.

## Assessment of the training intervention

### Study design

We applied a longitudinal design to assess the training intervention in the two counties. The first wave of data collection was at baseline (before training in supportive supervision) and the second one at end line (six months after training) from the same supervisors and CTC providers of health care.

### Sampling

We purposively sampled 16 CHEWs and three sub-County managers using the stakeholder sampling approach, where we identified participants who were involved in delivering the intervention and those who benefited from the training in supportive supervision. A total of 56 CHVs completed the supervision-tracking tool at baseline and 54 completed the tool at end line. Of these, only 34CHVs completed the Supervision Tracking Tool at both time points. Table 1 illustrates participants enrolled into this study at both baseline and end line.

**Table 1:** Number of participants who participated in the assessment of the training intervention in the Nairobi and Kitui counties.

		Baseline assessment (May 2015)					Endline assessment (December 2015)				
		Nairobi County		Kitui County			Nairobi County		Kitui County		
	Participants	Male	Female	Male	Female	Total	Male	Female	Male	Female	Total
Indepth-interviews (IDIs)	Sub-county community health service managers	1	1		1	**3**	1	1	1		**3**
	CHEW	2	-	1	-	**3**	2	-	1	-	**3**
	CHVs	4	4	4	4	**16**	2	6	3	5	**16**
	CHV team leaders	2	2	2	2	**8**	2	2	2	2	**8**
Supervision tracking tool	CHVs peer supervisors and CHVs	28		28		**56**	18		36		**54**

### Data collection

In depth interviews (IDI): We qualitatively assessed changes in supervision approaches using face to face IDIs with purposively selected CTC providers of healthcare (CHVs, CHV team leaders) and their supervisors (CHEWs and sub-county managers). These interviews explored perceptions of both supervisors and supervisees on the effect of the six-day supportive supervision training on supervision practices and frequency of supervision. We also explored supervisors’ perceptions towards the supervision checklists that they used to structure their supervision sessions as part of the intervention. IDI topic guides were translated into Kiswahili and then piloted in non-study sites. We recorded all interviews using digital audio recorders after obtaining written informed consent from participants. Time spent in the IDIs ranged from 30 to 45 minutes.

Supervision tracking tool: The Supervision Tracking Questionnaire (STQ) was used to quantitatively assess the frequency of supervision. The STQ was administered face-to-face at both baseline and end line, with purposely-sampled CHVs. The tool acts as a record keeping instrument for the number and type of supervision activities (i.e. group, one-on-one, and/or accompanied home visits) that supervisors had carried out in the previous three months, as well as the cadre of supervisor. The Supervision tracking Tool is in S5 File.

### Data management and analysis

Audio recordings of IDIs were transcribed into Swahili/Kamba in MS Word and translated into English by a team of research assistants. A team of seven qualitative researchers jointly developed a deductive analytical coding framework for the assessment based on findings from the context analysis. We used NVivo (Version 10) [28] for developing the framework and data coding. We pre-tested the analytical framework by jointly coding the data and making revisions until all coders had consensus on the themes in the coding framework. We used the thematic framework approach to analyze the qualitative data. We described each theme using selected quotes for illustration. New categories and themes were inductively added after reviewing IDI transcripts. We stopped analysis of qualitative data when we reached saturation, that is, when there were no more new emergent themes arising from the transcripts. Quantitative data were analyzed using SPSS (Version 24.0). Significant changes in the frequency of supervision across types of supervision sessions (categorized as either one-on-one, group, accompanied home visits) held across both districts were assessed using a Wilcoxon-Signed Rank Test. Missing data was handled using a list-wise deletion, resulting in only 34 cases retained for analysis. All tests were conducted for 95% confidence with α = 0.05.

### Ethical approval and consent to participate

Ethical clearance was obtained from the Kenya Medical Research Institute Ethics Review Committee (Non-SSC Protocol No 399 and Non-SSC Protocol No.144). We obtained administrative clearance to engage with health providers at sub-county and community levels from the Nairobi and Kitui County Health Departments and Health Management Teams.

## Results

The context analysis enabled us to establish how supervision of CTC providers of healthcare was taking place, who was responsible and the views of both supervisors and supervisees. Results from the context analysis are organized into the three principal themes: (a) supervision practices and perceptions; (b) plurality of supervisors and coordination; and (c) challenges faced during supervision. Findings from the assessment of the intervention have been presented thematically on perceptions of the effect of the training on supervision practices and on the frequency of supervision.

### Findings from the context analysis

#### Plurality of supervisors and lack of coordination

CTC providers of health care reported that a wide range of persons in the health system and community settings perceived themselves as having authority to supervise CHVs. Those whom they identified as supervisors of CTC providers were: community health committee (CHC) members, CHEWs, link-facility in-charges, the sub-county health management team, local government administration officers (chiefs) and CHV team leaders who were informally assigned supervisory roles by CHEWs. This often led to lack of clarity among CHVs on whose directions to follow. At times, the roles of members of CHCs, community leaders and CHEWs in problem solving in the community overlapped—e.g. when a community member complained about a CHV’s performance. CHEWs were not always the first point of contact for supervisory needs because they were considered as ‘outsiders’ by the local CHVs. This was despite their formal roles in the CHCs. *“*‥*you will find that the community health workers report directly to the community health committee*. *…the CHEW comes in as the last resort because we always believe that any problem that the community has*, *they should try and solve it themselves first is when like the outsider comes in…”* (CHEW, Nairobi). Some CHVs reported that they preferred being accountable to CHCs because CHEWs were at times not available to provide guidance. CHCs were active in supervising CHVs in Nairobi compared to Kitui and CHVs perceived them to have invested more time and effort in supervision. Consequently, CHVs considered CHCs as the first point-of-contact with regard to supervision, despite CHEWs being their official supervisors. Dual reporting to both CHEWs and CHCs resulted in complexity in supervision. Lack of coordination between the two resulted in disagreements and confusion in the community units.

#### Supervision practices and approaches

Most CHEW respondents reported supervising CHVs through monthly meetings, accompanied household visits and by reviewing monthly reports. They also used community dialogue and action days as opportunities to observe how CHVs performed as they delivered health education to community members. All supervisors in both counties reported that supervision sessions motivated CHVs. Guidance received from CHEWs during supervision sessions was the main source of motivation among CHVs. CHEWs from both counties stated that supervision from sub-county health managers was inadequate and this demoralized them. One key theme that emerged from the interviews with CHEWs was that they would be motivated if their sub-county level managers accompanied them during activities in the community, as illustrated by this quote:*“…they* (sub-county health management team) *also need come down there and meet the CHVs at least to give them hope because we are at the ground level*. *There they know I have my superior but they don’t usually see them…*.*the superiors need also to come and to put more strength*. *You know sometimes when a superior comes you feel you are recognized and you are more important”* (CHEW, Kitui County).

In most cases, supervision of CTC providers was reported to occur when there was a problem in the community unit and thus were perceived as faultfinding sessions. *“Supervision is only meant to do with trying to encourage those who might have chosen to relax a bit or those who are missing out on some point that is when you can go there and remind them on what is required of them so that they can correct on it”* (Policy Maker, MOH).

This fault-finding approach was also similar in both counties. CHEWs reported that they focused on correcting mistakes during supervision sessions than supporting CHVs to perform better and solve problems. This understanding of supportive supervision is illustrated in this quote: “*…they* (CHVs) *are supervised there by the community health committees from the same villages*. *Ours is just to go there to assist or give them what we call supportive supervision*. *That is*, *we support them where they are not performing*, *where they are going wrong*. *We tell then ‘no these are the right directions’* (CHEW, Kitui County).

#### Challenges faced during supervision of CHVs

Heavy workload was reported by CHEWs as being the main barrier to supervision. We found that CHEWs were supervising a large number of CHVs, which affected their ability to provide oversight. In some cases, CHEWs were reported to oversee up to two community units as one responded stated: “*Our CHEW is very committed because (*he/she) *monitors two community units that are far from each other*‥*”* (CHV, Nairobi County). CHEWs who doubled up as primary health facility staff faced conflict between undertaking facility roles and supervising CHVs. These dual roles negatively affected the frequency of supervision by CHEWs. In the event of a conflict between these two roles, CHEWs often prioritized the facility roles:*”I am afraid of closing the facility then patient dies back here when I am doing supervision”* (CHEW, Kitui County). This dual role affected their efficiency as CHEWs, as stated by one CHEW: “*So far I have not held any household visits because*, *I don’t have that time since I work in the lab and most of the time I am in that facility*. *What I do is that I just supervise them from the lab”* (CHEW, Kitui). Additional challenges in supervision of CHVs are summarized in Table 2.

**Table 2:** Summary of key gaps and challenges in supervision identified during the context analysis.

	Supervision gap	Description	Quote
**Health system challenges**	CHEWs who had a clinical background focused their efforts in running facilities and invested little or no time supervising CHVs in the community	Most of the CHEWs in the study sites had a clinical background (nurses, laboratory technicians, medical engineering and medical records). They did not prioritize CHV work and tended to focus their time on facility-based activities and ignored community health.	*“For instance we have laboratory technicians; we have the medical engineers*. *A medical engineer has been trained to maintain the medical equipment’s in the facility*, *that is the background of the work and you have employed as a community health extension worker”* (**Policy Maker, MoH)**.
	Dual roles of supervisors	Most CHEWs were employed with a clinical professional background were assigned clinical roles in the link facilities and still expected to function as CHEWs. This increased the workload and negatively affected how they supervised CHVs	*“As I am the nurse here and I am also the CHEW*, *so you find that they may have planned for a supervision on a certain day but on that day*, *the hospital is very busy*. *So I cannot close up the hospital and go for the supervision”* **(CHEW, Kitui County)**
	Inadequate inputs for Supervision	CHEWs reported that they lacked supervision checklists to provide guidelines and standards. They reported this as an impediment in supervision. Supervisory guidelines were only reported to be available where vertical programs no defined standards	*“Also*, *we don’t have the supervision tools for supervision; we just go to the ground to supervise”* **(CHEW, Kitui County)**
		The most frequently reported logistical challenge for supervisors was lack of transport.	*“Here I once had my small bicycle*, *but now the roads are very bad you can’t access some areas and it becomes really tiresome using the bicycle*. *A motorbike would be much better”* **(Facility Manager, Kitui County)**.
**Supervisor challenges**	Inadequate problem solving skills	CHEWs were sometimes be caught in conflicts between the CHVs and community members. Most of them lacked skills to resolve these conflicts.	*“If it’s between them and the community we just encourage them because we (CHEWs) cannot summon a member of the community and tell them the mistake they did”* **(CHEW, Kitui County)**
**Cultural challenges**	Cultural barriers (Age)	Age was a factor that affected interaction between the CHEWs and CHVs. A supervisor’s age in relation to the supervisees affected whether the supervisor would be able to provide adequate supervision.	*“I’m limited especially those (CHVs) old ones*. *They will shout at you*. *They tell you ‘I’m old you can't tell me anything’* **(CHEW, Nairobi County)**

### Results from the assessment of the training intervention

While assessing the supervision training intervention, we measured its effect on the supervision practices that were documented in the context analysis and on the frequency of supervision. We present both qualitative and quantitative findings here to supplement each other.

#### Perception of supervision practices and approaches

Overall, the intervention appeared to have had a positive effect on supervision practices such as mentoring and problem solving. From the IDIs, supervisors reported that before the training supervision was hierarchical and top-down. The training on supportive supervision encouraged them to change their approach and incorporate dialogue, creating a conducive environment for teamwork. As one CHV team leader explained: *“…then*, *we were using orders*, *so instead of orders*, *it is dialogue*, *instead of forcing*, *it is agreeing*. *And also we do share*, *before we do anything*. *If there must be something to talk about*, *so we talk about it and be in the same journey*.*”* (CHV Team Leader, Nairobi).

This change in supervision was also highlighted by CHEWs who supervised CHVs: *“That training was good because it taught us on supervision because it is something that we didn’t understand before*, *we had never been taught especially me*, *I had not understood*. *I used to know that supervision is when we have an action day*, *or dialogue*, *I didn’t know that sometimes you take your own time to go and follow up on the CHVs*, *and also I didn’t know that when you are following someone*, *there are times that you give feedback*, *I just knew that it was just that way*.*”* (CHEW, Kitui).

Supervisors consistently expressed their intention to make supervision sessions as supportive as possible by considering the welfare of their supervisees and enhancing supervisees’ capacity in instances when they did not know how to handle certain situations in the course of their work. Firstly, accounts from CHEWs point out that they used monthly group supervision sessions to encourage and motivate CHVs: *“The supervision is about encouragement so that the* [site name] *group is going on well with incentives or not…”* (CHEW, Kitui). Monthly group supervision sessions were held in the local primary health facility. CHEWs provided monthly group supervision as part of these meetings where CHVs submitted their service delivery records for summarization by the CHEW.

Secondly, supervisors utilized supervision sessions to mentor and coach their supervisees. Interviews with CHVs suggested that they appreciated these efforts and they viewed them as important in improving the way they worked *“…*‥*so she encouraged me to continue doing the things that I had done the right way and she also corrected me on what she thought I was not doing right like the way I was knocking and calling out some people might find it disrespectful*.*”* (CHV, Kitui). There was an indication that supervisors made conscious efforts to mentor and coach their supervisees as they provided supportive supervision:

*“The last supervision I did was meant to lead by example because the team leaders who I supervise were also going to supervise CHVs so I supervised them and from that supervision* [session] *they learnt how supervision is being carried out and it was like an example to them so they are going to use this to supervise*, *the skills they picked or the way they saw the supervision being conducted …”*

(CHEW, Nairobi)

Thirdly, supervisors utilized supervision sessions as forums for information sharing between them and the supervisees. For most of the CHVs, these sessions also provided opportunities for discussing solutions and support related to health problems in the community, as one CHV stated: *“…*.*when we get to the meetings she asks us what challenges we have faced and what good things we have experienced and we share*. *If there is something that happened in the hospital*, *she reports to us as the team leaders*. *For instance the polio campaign she would tell us about it and then she would ask if you have a patient that is severely sick and you probably need the doctors to come and see that person at home so we discuss such things*.*”* (CHV Team Leader, Nairobi).

Accounts from IDIs indicate that developing skills in supportive supervision was a strong motivating factor for both supervisors and supervisees in study sites where supervision was not disrupted due to devolution processes. *“…*‥*the CHVs* [peer CHV supervisors] *who are trained*, *now have the knowledge on supervision unlike earlier where we had like dictatorial kind of supervision*. *Now we have a soft approach*, *also now they know what they are looking for*, *also there is kind of motivation*. *You find that the CHVs feel that now somebody is looking at our work so they have to do good work”* (Sub-County CHS Focal person, Nairobi).

Possessing skills in supportive approach also had an effect on the satisfaction of the supervisors, especially when their supervisees heeded the guidance provided, as one CHS focal person opined:

*“…when you are supervising you are like a mentor*. *You also mentor those you are supervising*, *especially now we are talking of supportive supervision*, *not the previous supervisions*, *when people went to …to look for the wrongs*. *Today we support while supervising…You also feel some satisfaction if this person heeds your advice*.*”*

(sub-County CHS Focal Person, Kitui)

The supervision checklists that we provided to the supervisors as part of the intervention were helpful in facilitating the supervision process. CHEWs that were interviewed perceived a marked improvement in the way they supervised CHVs after receiving supervision checklists in the course of the intervention.

***“****The supervision tool is systematic; it has a way of reminding me what to take when am in the process of a group formation for example recording the attendance*, *making sure I touch on the three key areas supportive administrative and educative*. *Before I got the supervision training I just did it randomly*, *we did not record any minutes at that time and we had nobody to do follow-ups but as the months continued after getting the trainings*, *supervision improved I could now use the tool I could supervise them and give the responsible people the follow ups*.*”*

(CHEW, Nairobi).

#### Frequency of supervision among CTC providers of healthcare

Results from the Wilcoxon Signed Rank Test comparing the differences in frequency of three different types of supervision (one-on-one, group, accompanied home visits) suggests that the intervention was associated with a statistically significant change in supervision frequency in both accompanied home visit (Z = -2.719, p<0.05, r = .054) and group (Z = -2.442, p<0.05, r = 0.49) supervision in Kitui only. Whereas the frequency of group supervision significantly decreased, the frequency of accompanied home visits supervision significantly increased (Table 3).

**Table 3:** Results of Wilcoxon Singed Rank Test comparing type and frequency of supervision before and after the introduction of the supportive supervision training.

Study county	Type of Supervision	Time	n =	Z	p-value
**Kitui**	One-on-one supervision	Pre-intervention	26	-.939^b^	0.348
		Post-intervention	26		
	Group supervision	Pre-intervention	26	-2.442^a^	0.015*
		Post-intervention	26		
	Accompanied home visits	Pre-intervention	26	-2.719^b^	0.007*
		Post-intervention	26		
**Nairobi**	One-on-one supervision	Pre-intervention	8	-.743^a^	0.458
		Post-intervention	8		
	Group supervision	Pre-intervention	8	-.577^b^	0.564
		Post-intervention	8		
	Accompanied home visits	Pre-intervention	8	-1.890^b^	0.059
		Post-intervention	8		

^a^Based on positive ranks

^b^Based on negative ranks

*<0.05

Findings from qualitative data suggest that supervision became more frequent in some sites and that supervisors used the supervision checklists that were provided at the beginning of the intervention—as one CHEW reported: *“…supervision before was just random without any tool and any procedure*, *but … now it has become routine and as before it’s just maybe one*, *we get one supervision now*, *the next one in two weeks the next one in a month*, *at least now its constant and my supervisor is using a tool…”* (CHEW, Nairobi).

One respondent attributed positive changes in supervision to a sub-County CHS coordinator who had been was implementing the principles of supportive supervision that were taught during the training. *“Yes* [there is a change], *because at this one depends with the community coordinator we have around because the last times we could even go a year or even without supervision”* (CHEW, Nairobi).

In Kitui County, supervision was disrupted by the devolution process: *“There is no form of supervision although sometimes we sit down in the office and discuss and my supervisor is supposed to be [Name Omitted]* (CHEW, Kitui). Another CHEW, who sounded frustrated, said: *“I don’t feel good because I know there is no work that has no supervision*. *We do this work voluntarily but we still need supervision to make yourself better and it makes you feel like you are working when someone rectifies you and tells you what you are doing right and what you are doing wrong then you feel encouraged but currently there is no supervision*.*”* (CHEW, Kitui).

Of interest is that a sub-county level supervisor stated that community health activities in one of the community units were revived after implementation of the supportive supervision intervention despite having a reduction in the frequency of supervision sessions after the training intervention. *“… I see that there are changes*, *let me give an example of [name of community unit withheld]*. *…before*, *eh the community units never used to be that active… Because they did not have any hope… In fact surprisingly majority of them are even reporting*, *their reports are here*.*”* (sub-County CHS Coordinator, Nairobi).

## Discussion

Our study revealed significant challenges in the way supervision of CTC providers of health care was coordinated among the different actors at community level in Kenya. We observed this in the way formal (CHEWs) and informal (local chiefs and CHCs) supervisors carried out supervision. Multiple supervisors with limited skills in supervision diminished the value of supervision. Policy guidelines on supervision of CTC providers of healthcare in Kenya are also not clear. Operating guidelines for CHCs do not explicitly state their role in supervision of CTC providers of health care. This overlap in responsibility between local chiefs (extension of central government), community level administrators (part of county administration) and CHCs further complicates the picture [29, 30]. The roles of sub-county level actors in the coordination of supervision is often unclear. For sub-county level managers to set up coordination mechanisms for supervision of CTC providers of healthcare, they need to be sensitized on the importance of community health services in achieving both national development goals and universal health coverage [9].

Inadequate training of supervisors on supervision, lack of supplies for supervision and logistical challenges contribute to demoralization of supervisors of CTC providers of health care. As demonstrated by Ndima *et al*. (2015) in a similar study in Mozambique, this lack of adequate support led to demoralization of supervisors and poor performance [20]. A systematic review by Kok *et al*. (2014) documents that providing logistical support to supervisors to facilitate frequent supervision and continuously updating their supervision skills has a positive relation to better performance of CTC providers of healthcare [31]. For sustainability of effective supervision of CTC providers of health care, there needs to be mechanisms in place to ensure regular and continuous capacity building of supervisors. Within our study, supervisors appreciated the supervision checklist tools and felt that the tool helped improve their supervision practices. This is in keeping with previous research, which revealed that supervision guidelines can fill the gap between actual and recommended practice and also to enhance consistency of supervisory practice [32].

There is limited empirical evidence on the effect of increasing the frequency of supervision. Regular supervision on its own has been found to be insufficient to improve the productivity of health workers [33]. Marquez *et al*. (2002) argued that supervision interventions may be effective during the early stages but are difficult to sustain due to factors such as staff transfers, changes in health system and individual health workers factors[21]. Because of devolution, there were widespread changes for staff during the study period, including re-assignment of CHEWs and restructuring of the community health approach, depending on the local county context and priorities. These uncertainties affected the frequency of supervision sessions in our study sites. A recent study in Kenya found that CHEWs were felt to have an increased workload following devolution, since a number of counties have sought to increase community health service coverage by recruiting new CHVs without increasing the number of CHEWs to supervise the greater number of CHVs—which has implications for the quality of supervision [34].

In one of the study sites, supervision was seen to revive activities in a community unit that was considered not to be functioning. This finding is in line with those of an experimental study documented by Loevisohn *et al*. (1995), which found a direct correlation between the supervision and performance of primary health care workers in the Philippines. However, enhanced performance is only achieved if the supervision activities are productive and seek to improve performance of the supervisees [35].

The Kenyan REACHOUT context analysis found that supervisors mainly conducted supervision when there was a problem [18]. Supervisors also used supervision to find faults in CHV performance. This approach has been shown to create dissatisfaction among CTC providers of health care. A study by Gopalan *et al*., (2012) in India revealed that dissatisfaction with supervision among CTC providers of health care led to lower intrinsic job motivation and reduced community recognition [10]. Our training intervention had a positive effect on the approach to supervision, shifting from fault-finding to supportive. Consistent with the literature is that CTC providers of health care reported positive changes in the way that their supervisors provided supervision and feedback six months after implementing the supportive supervision intervention. Similarly, accounts from CHEWs and CHVs during the IDIs in our study suggest that constructive interactions and feedback with a supervisor created a sense of job satisfaction and motivation. Various authors such as Jenkins *et al*. (2013) and Oliver *et al*. (2015) have documented that supervision approaches that are supportive to CTC providers of health care can ensure high quality of work, motivate them and create a sense of legitimacy [4, 36]. Our qualitative findings confirm the importance of supportive supervision on motivation of CTC providers of health care. CHVs reported being more motivated when supervision was done in in a supportive manner. A similar finding was reported in Madagascar, where CTC providers who viewed supervision as inadequate reported being demotivated [8, 14] compared to those who received adequate supportive supervision.

Peer supervision (conducted by CHV team leaders) was another form of supervision that we observed in our study sites. Hill *et al*. (2014) suggest that this approach shows the most potential in improving the performance of CTC providers of health care because it takes advantage of peer-peer empathy to facilitate learning, support and problem solving. Peer supervisors in our study also reported changing their approach to supervision from administrative to supportive during the in-depth interviews. This is promising evidence that peer supervision can be effective and can be formally recognized as an alternative form of supervision. The Kenyan MoH does not formally recognize the role of CHV peer supervisors, although peer supervisors are elected by CHVs to represent them and provide oversight to fellow CHVs. A study in Rwanda showed that peer supervision of CTC providers was critical is improving their performance, motivation and collaboration [37].

Joint problem solving and action planning are features of supportive supervision that motivated CTC health providers in this study. Interestingly, a systematic review by Bosch-Capblanch *et al*. (2008) pointed out that inasmuch as problem solving and feedback are recognized as key for improving health worker performance, they are not prominently featured in supervision programs [38]. Respondents from our IDIs described a positive relationship between feedback, joint problem solving and motivation.

Supervisors and CTC health providers in our study demonstrated that developing technical capacity in supervision and providing them with checklists to facilitate supportive are a source of motivation. As much as checklists are important for structuring supervision, they may encourage authoritarian and inspection or unsupportive approaches to supervision [38]. There needs to be a balance between using checklists as a tool to provide structure to supervision and controlling approaches to supervision.

## Strengths and limitations

Findings from this study were informative and useful in characterizing the factors that influence supervision of CTC health providers in Kenya. The study further demonstrates the effect of training supervisors in supportive supervision on the supervision practices and frequency of supervision. There were however inherent limitations in the study:

- First, the investigators encountered selection bias since in some cases the CHEWs or sub-County focal persons selected CHVs to participate in the IDIs. It is possible they may have selected close associates, the most eloquent respondents or persons with whom they had a good working relationship rather than a representative sample.
- Second, social desirability bias may have occurred because CTC health providers were afraid of speaking about the weaknesses of their supervisors in supervision, communication, among others.
- Third, it was not possible to verify some responses provided by the participating CTC health providers such as the number of supervision meetings that had been conducted since the intervention. This was because we conducted most of the interviews in community settings where supervisors of CTC providers did not have access to minutes that were stored in the link primary health facility. Therefore, we could not access all minutes of supervision meetings
- Fourth, we conducted this study during the period immediately following the introduction of national devolution reforms. This revealed the varied appreciation for and investment in community health services between county governments [39]. In particular, Nairobi county changed their staffing policy, leading to health workers recruited as CHEWs prior to devolution being re-distributed to other positions, with no clear plans to replace them. Kitui County restructured the community health approach, changing roles and responsibilities for CHVs and CHEWs, in order to better meet the needs within their local context. These changes created uncertainty among health workers and CTC providers, with varied implications for implementation of community health services. In addition, McCollum *et al*., (2017) found that where county governments prioritized to invest in community health, the emphasis was typically on expanding coverage, rather than strengthening quality and supervision [34]. This may have introduced bias to our findings on frequency of supportive supervision due to insufficient operational support for supervision by the study counties.
- Finally, the end line data collection was conducted six months after implementation of the intervention; this may not have been sufficient implementation period to make a measurable impact. We also had to comply with the multi-country project timelines that required the assessment to be completed in December 2015.

## Conclusions and recommendations

The supportive supervision intervention had a positive effect on the supervision practices, helping supervisors shift from fault-finding to more supportive supervision. Supervisors took up the skills that were imparted to them during the training and attributed these changes in their supervision practices to the training they received. In view of these findings, recommendation by authors are summarized in Box 1:

**Box 1. Recommendations based on the findings of the study**

1. Training of supervisors of CTC health providers of health care on supportive supervision needs to be scaled up in all counties to ensure there is sufficient technical capacity in supportive supervision.
2. County Health Departments need to recognize the vital role that supportive supervision plays in providing quality health services and therefore need to factor in logistical support and supplies for supervision in the annual county public expenditure estimates.
3. County Health Departments need to formally recognize the important role that peer supervisors play in supervision of CHVs. This form of task-sharing may enhance supervision by ensuring that CHEWs, the formal supervisors, are supported with the administrative roles of supervision by peer supervisors.

## Supporting information

S1 File (PDF)

S2 File (PDF)

S3 File (PDF)

S4 File (PDF)

S5 File (PDF)

S6 File (SAV)

S7 File (ZIP)

S8 File (ZIP)
