# Supplementary material for: Supportive supervision of close-to-community providers of health care: Findings from action research conducted in two counties in Kenya
Source: PLoS One. 2019 May 29;14(5):e0216444. doi: 10.1371/journal.pone.0216444 (PMC6541245; doi:10.1371/journal.pone.0216444)
Supplement: S1 File — (PDF) [file pone.0216444.s001.pdf]

Reachout
Linking Communities & Health Systems

Group Supervision Checklist

Who and When: Tool to be completed by CHEW during supervision of CHV(s) at group supervision meetings. The tool is considered complete once the CHEW has given the CHV feedback and all parties sign the document signaling that a meeting has taken place.

NB: The tool should be accompanied with a signed attendance sheet.

Prior Preparation: The CHEW needs to consider the following before completing this tool:

1. Meeting agenda, see guidance below of what agenda might entail
2. CHEW should have knowledge of the catchment area assigned/ no. of households assigned to CHVs
3. Prior feedback about the CHVs' work from observations made by the CHEW and/or from reports given by others e.g. community, dialogue days, health facility staff, or the CHV(s)' peers.
4. The CHEW should have a summary of the routine data for presentation to the group.
5. Group supervision should be carried out after a dialogue day in the event that the two occur in the same month
6. Prior appointment with CHVs to allow for meeting preparation
7. Educative material to teach during the session

**Link Health Facility(ies) Name:** *(write all if more than one unit involved)*	**Date:**
**Community Unit(s) Name:** *(write all if more than one unit involved)*	**Time meeting started:**
**County:**<br><br>**Sub-County:**	**Time meeting finished:**
**CHEW's Name:**	

Group Supervision structure (Half day)
Document key points at section C

- Present agenda for the meeting
- Begin with issues arising from peer supervision meetings that have happened
- Discuss matters arising from last group supervision meeting and the status of action points which were to be followed up
- Discuss dialogue days i.e. planning for the next one if not conducted yet e.g. how to mobilize and agenda. If one has been done, discuss problems identified at dialogue day, activity plans and their status.
- Discuss other activities conducted in the month and matters arising e.g. action point, status. This can include action days, outreaches, and training.
- Have an activity on experiences from the unit in the past month – ask three participants to share both a positive and a challenging story they have experienced in their work.
- Present a summary of routine data collected in the month. Discuss data findings with the CHVs.
- Discuss monthly reporting i.e. data quality, frequency of reporting
- Discuss referrals i.e. documentation and challenges
- Discuss home visits conducted in the week e.g. percentage of conducted by CHVs, matters arising from observations by supervisors, general action points
- Provide feedback of the program from others e.g. health facility staff, community, CHC meetings, DHMT
- Educative session. Do a participatory refresher training on a CHV task, responsibility or problem area (About 1 hour). Sample education topics available from supervision trainers if required.

C: Meeting Documentation
NB: Action points should be clear defined it terms of what the action is, who is responsible for the activity, and when it is supposed to be done.

Supportive Function<br>Key issues discussed with the CHVs

Problems and Barriers Identified:

Action Points:

Administrative Function
Key issues discussed with the CHV(s)

Problems and Barriers Identified:

Action Points:

Educative Function

Which topic(s) did you capacity build the CHVs on?

Problems and Barriers Identified:

Action Points:

**CHEW's Signature:**	**Date:**

Attendance sheet

CHVs Name and Unit	Signature
