# Supplementary material for: Supportive supervision of close-to-community providers of health care: Findings from action research conducted in two counties in Kenya
Source: PLoS One. 2019 May 29;14(5):e0216444. doi: 10.1371/journal.pone.0216444 (PMC6541245; doi:10.1371/journal.pone.0216444)
Supplement: S2 File — (PDF) [file pone.0216444.s002.pdf]

Reachout
Linking Communities & Health Systems

One-on-One and Group Supervision Checklist

Who and When: Tool to be completed by CHEW during supervision of CHV(s) on one-on-one meetings; after observing a CHV conducting a home visit; and during group supervision meetings. The tool is considered complete once the CHEW has given the CHV feedback and both parties sign the document signaling that a meeting has taken place.

NB: The tool should be accompanied with a home visit observation checklist if completed after a home visit; and the tool should be accompanied with a signed attendance sheet if completed during a group supervision meeting.

Prior Preparation: The CHEW needs to consider the following before completing this tool:

1. Meeting agenda, see guidance below of what agenda might entail
2. CHEW should have knowledge of the catchment area assigned/ no. of households assigned to CHV(s)
3. Prior feedback about the CHV(s)' work from observations made by the CHEW and/or from reports given by others e.g. community, dialogue days, health facility staff, or the CHV(s)' peers.
4. For group supervision the CHEW should have a summary of the routine data for presentation to the group.
5. Group supervision should be carried out after a dialogue day in the event that the two occur in the same month
6. Prior appointment with CHV(s) to allow for meeting preparation
7. For one-on-one supervision, the CHEW should have a home visit observation checklist and a spot check report if the CHV or their area of work was observed in order to provide feedback to the CHV and/ or to discuss action points that arose from the observation/ spot check

**Link Health Facility Name:**	**Date:**
**Community Unit Name:**	**Time meeting started:**
**County:**<br><br>**Sub-County:**	**Time meeting finished:**
**CHEW's Name:**	
**CHV's Name:**	

One-on-one Supervision Structure

Document key points at section C

- Begin with asking the CHV about their general welfare to find out if there are issues affecting their work that they need help with e.g. personal issues affecting work (illness, family matters, stress); work issues affecting person (burn out, stressful issues encountered in the field, work load, competencies)
- Find out whether CHV attended any group supportive supervision sessions
- Discuss home visit observation/ spot check if it was done for the CHV and action points from it
- Discuss their monthly reporting i.e. data quality, frequency
- Discuss referrals i.e. documentation and challenges
- Discuss community engagement activities done e.g. mobilization for outreach, concerns raised by community
- Provide additional feedback

C: Meeting Documentation

NB: Action points should be clear defined it terms of what the action is, who is responsible for the activity, and when it is supposed to be done.

Supportive Function<br>Key issues discussed with the CHV<br><br>Problems and Barriers Identified:<br><br>CHV has attended group supportive supervision sessions?<br><br>Action Points:<br><br>*Administrative Function*

Key issues discussed with the CHV

Problems and Barriers Identified:

Action Points:

Educative Function

Which topic(s) did you capacity build the CHV on?

Problems and Barriers Identified:

Reachout
Linking Communities & Health Systems

Action Points:

**CHV's Signature:**	**Date:**
**CHEW's Signature:**	**Date:**
