# Supplementary material for: Supportive supervision of close-to-community providers of health care: Findings from action research conducted in two counties in Kenya
Source: PLoS One. 2019 May 29;14(5):e0216444. doi: 10.1371/journal.pone.0216444 (PMC6541245; doi:10.1371/journal.pone.0216444)
Supplement: S3 File — (PDF) [file pone.0216444.s003.pdf]

CHEW SUPERVISORY SPOT CHECKS

Background:

The CHEW will use this document to enable documentation and assessment of spot checks done in the field.The documentation will enable follow-up of issues observed and of action points in order to improve quality of services. Spot checks can be done for an area covered by one CHV or more than one.

Preparation:

- Community's awareness of CHEW. The CHEW should be known to the community members so that he/she is not viewed with suspicion
- A CHEW should arrange to make the spot checks with a CHC member who is known to the community. If a CHC member is not available, the CHEW can conduct the spot checks with a CHV
- The CHEW should have completed Service Delivery Logbooks from CHVs to confirm during the spot checks e.g. nos. of pregnant women identified, referrals done and uptake, identified defaulters, adoption of healthy practices e.g. clean up activities.
- The CHEW should be ready to take feedback from community members about the program and the CHVs during the spot checks. Feedback will be achieved directly e.g. through asking and listening or indirectly by observing

Feedback: Feedback for spot checks should be provided to CHVs working in the observed areas and can also be an agenda at a planned one-on-one supervisory meeting.

**Link Health Facility Name:**	**Date:**
**Community Unit Name:**	
**County:**<br><br>**Sub-County:**	
**CHEW's name:**	
**Position of person conducting spot checks with CHEW e.g. CHV, CHC member:**<br><br>**Name of the person:**	
**Area (s) where spot check (s) was conducted:**	

A: Documentation of Spot Check

Nos. of households visited by CHEW during the spot check: [ ]

Nos. of households confirming visit by CHV within the past two months: [ ]

Are all the households visited by the CHEW registered?

………

………

Are all members in the households registered?

………

………

**Comments from households about CHVs work and recommendations for the program**

Confirm data collected by CHVs on service delivery log books for the selected households e.g. referrals, whether they took up referrals, nos. of pregnant women, etc.

General Observation of the Community by the CHEW e.g. Water, Sanitation and Hygiene issues, general health condition of the community and potential health threats

Reachout
Linking Communities & Health Systems

B: Feedback to CHV(s)

Key issues discussed:<br><br><br>Barriers and problems identified:<br><br><br>Action Points:

**CHV(s) Name and Signature(s):**	**Date:**
**CHEW's Name:**<br><br>**Signature:**	**Date:**
