# Supplementary material for: Supportive supervision of close-to-community providers of health care: Findings from action research conducted in two counties in Kenya
Source: PLoS One. 2019 May 29;14(5):e0216444. doi: 10.1371/journal.pone.0216444 (PMC6541245; doi:10.1371/journal.pone.0216444)
Supplement: S4 File — (PDF) [file pone.0216444.s004.pdf]

Reachout
Linking Communities & Health Systems

Home Visit Observation Checklist

Who and When: Tool to be completed by CHEW during supervision of CHV(s) during an observed home visit. The tool is considered complete once the CHEW has given the CHV feedback and both parties sign the document signaling that a meeting has taken place.

Prior Preparation: The CHEW needs to consider the following before completing this tool:

1. CHEW should have knowledge of the catchment area assigned/ no. of households assigned to CHV(s) so as to ensure observations are spread out and not done for the same households
2. Prior appointment with CHV(s) to allow for meeting preparation
3. Prior permission should be sought from the household members

Guidelines

- Have the CHV introduce you to the household members and thank them for having you
- Explain the purpose of your visit: To identify supervisory support areas which will ensure CHVs provide services that are satisfactory to the community
- Explain the need to take notes for follow-up and assure household of confidentiality
- Allow the CHV to conduct the visit as if you are not there

**Link Health Facility Name:**	**Date:**
**Community Unit Name:**	**Time observation started:**
**County:**<br><br>**Sub-County:**<br><br>**Household Observed:**	**Time observation finished:**
**CHEW's Name:**	
**CHV's Name:**	

A: Meeting Documentation

Key

0 = Not done

1 = Attempted with little success

2 = Achieved fairly

3 = Achieved successfully

N/A = Not applicable

**Aspects of service being observed**	**Score**	**Comments**
Rapport with household members		
Outline visit purpose and seeks consent		
Assurance of confidentiality		
Follows up on issues raised by household at previous visit e.g. uptake of referrals		
Uses log book as checklist and covers all stated areas		
Asks about each individual household member separately		
Gives household members time to talk and listens to them		
Invites household members to define problems and guides them on possible solutions		
Provides referral for ANC		
Identifies home deliveries and refers appropriately		
Provides advice on <5 years children i.e. breastfeeding and immunization and refers		
Identifies immunization and ANC defaulters by confirming at Mother and Child Booklet/ Clinic care		
Provides advice on family planning and refers appropriately		
Identified sick household members and refers e.g. those with fever, diarrhea, cough, injuries		
Assesses and advises on prevention of Malaria		

Reachout
Linking Communities & Health Systems

Aspects of service being observed	Score	Comments
Assess and advises on hygiene and sanitation at home		
Advises on proper nutrition		
Identifies those on TB and ART treatment and follows up on their adherence to care through assessment of clinic cards, pill count, etc.		
Provides Home Based Care knowledge to care givers of the ill		
Uses job aids to support information dissemination		
Asks for additional questions and concerns		
Hands out referrals form to household members		
Provides IEC materials		
Double checks the log book for completeness		
Thanks household members and agrees on next visit		

B: Discussion of task that has just been observed

- Assess CHV's service delivery log book for completeness
- Arrange meeting with CHV
- Discuss observed task as per the observed aspects

Key Issues Noted:<br><br><br><br>Action Points:

Reachout
Linking Communities & Health Systems

**CHV's Signature:**	**Date:**
**CHEW's Signature:**	**Date:**
