# Supplementary material for: Supportive supervision of close-to-community providers of health care: Findings from action research conducted in two counties in Kenya
Source: PLoS One. 2019 May 29;14(5):e0216444. doi: 10.1371/journal.pone.0216444 (PMC6541245; doi:10.1371/journal.pone.0216444)
Supplement: S5 File — (PDF) [file pone.0216444.s005.pdf]

Supportive supervision of close-to-community providers of health care: findings from action research conducted in two counties in Kenya

Supplementary Information
Data Collection Tools

Contents

1. !n-depth Interview Guide: CHEW Level (English) 1
2. In-depth Interview Guide: Community Health Volunteer Level (English) 5
3. In-depth Interview Guide: Community Health Volunteer (Swahili) 9
4. Supervisor In-depth Interview Guide: Supervisor Level (Non CHEWs) - English 12
5. Supervisor In-depth Interview Guide: Supervisor Level (Non CHEWs) - Swahili 15
6. Supervision tracking questionnaire 18

1. !n-depth Interview Guide: CHEW Level (English)

Baseline and End line Evaluation

1. Work Background

a. Please tell us about your tasks and activities as a supervisor in the community health strategy

Probe: Whether they have dual roles and how they work around this e.g. working for different programs and organizations, facility vs. community work.
If CHW or CHC member ask how they juggle between volunteer work and their other occupations

b. What do you enjoy about your work as a supervisor?

c. How happy/ unhappy do you feel to do your work as a community health services provider?

Probe financial incentives; non-financial incentives (material e.g. bicycle / mobile phone or intrinsic, e.g. job satisfaction, impact of work on the community)

Probe for equipment and supplies, workload, working environment, communication, equipment and transportation, safety and sexual harassment, career perspective, community, clients, colleagues, other health workers.

d. How long/ how many years do you intend to work as a community health services provider?

Probe how come? How do you feel about your career prospects and skills development? What will influence how long you intend to stay doing this work?

e. What recognition do you receive for your work?

Probe how this influences motivation or job satisfaction.

Probe recognition from the community / family / organization

2. Supervision

a. Please describe how you have been supervised in your work in the last twelve months?

Probe: who (within and outside the Ministry of Health, etc.) and for each ask how often did it occur; what did it entail e.g. observation, group meetings, one-on-one meetings; what type of feedback they received

Probe: When was the last time they were supervised and what happened

b. What changes have been there in the past twelve months in how you are supervised?

Probe: How, what, how come – probe for directives given by supervisors, changes at practice level e.g. ask about changes in referrals, ask about changes in community dialogue days.

Probe: How do you feel about these?

c. What do you feel about the supervision you have received in the last twelve months?

Probe: Did it address your problems and how? What skill development occurs at supervision? How is feedback about your work given to you?

d. How has your work been coordinated in the last twelve months between your different supervisors within the program and others from outside?

Probe: Was there coordination? How did this occur? What impact did it have on your work (positive/ negative? Did it affect your motivation? How/How come?

Probe: On coordination for referral and community involvement

e. How could the supervision be improved to motivate you further

f. Kindly describe how you have supervised CHWs in the past twelve months?

Probe: With who, How, what, how come – probe for directives given to CHWs, changes at practice level e.g. ask about how they supervised e.g. meetings, one-on-one; ask about frequency of supervision; observed practice

Probe: How do you feel about these?

g. How frequently did you meet with CHWs/ CHEWs in the last twelve months and what did these meetings entail?

Probe: ask for frequency of meetings; type of meetings e.g. one-on-one, group, observation; who facilitated e.g. CHEW, CHCs, peer CHWs, etc; agenda of meetings

h. Describe briefly the new tasks and/ or new work tools you were given in order to be able to carry out supervision

Probe: barriers and effectiveness of use of supervision checklists and forms, carrying out group supervision, conducting supportive supervision, execution of problem solving and advocacy plans

i. What do you think the supervision you gave in the last 12 months was meant to achieve?

3. Referral

a. How did referral occur from the community to the facility in the past twelve months?

Probe: Was there a follow up process and how? What were the challenges of the process e.g. availability of referral forms, availability of services at facility, community expectations in the referral process

b. Describe briefly the new tasks and new work tools that the CHWs were given in order to be able to follow up on referrals

Probe: use of referral tracking register, process of following up on referrals

c. How do you think referral could be improved?

4. Community Engagement

a. How have you been interacting with the community in your work aside from supervising CHWs/ CHEWs in the past twelve months?

Probe: frequency of interaction, where, how it was planned, what interaction entailed

b. How has the community you work in been participating in your supervision work in the past twelve months?

Probe: Frequency and type of participation, where, if voluntary, who facilitated the participation and how

Probe: Role of Community Health Committees, presence of dialogue days and action days and how they were planned

c. What has been your role in enhancing community participation in the past twelve months?

Probe: Challenges faced in carrying out the role?

5. What worked and what didn't?

a. What impact did the HaQIQA intervention have on supervision, referral and community engagemant?

Probe: For each separately - Explore barriers and enablers

b. What aspect of the HaQIQA intervention did you find most effective?

Probe: How come? Why not?

c. Did you encounter difficulties in carrying out your new tasks?

Probe: Did you overcome them? How? How might you overcome them?

d. What other steps could you have taken to make HaQIQA effective?

6. Explanations

a. Can you suggest the reasons behind any improvements/changes in supervision, referral, and community engagement in the past twelve months in the community health unit you work in?

Probe on areas respondent identified before as evidence for change
Probe on whether there is a link to the HaQIQA intervention or not and ask for evidence of linkage

b. What enabled the changes brought about by HaQIQA to be/ not be consistently implemented as required?

c. Were there any other positive effects of the change implemented for you and your work that were not expected?

d. Were there any negative effects of the change implemented for you and your work that were not expected?

Probe for comments from clients (women and men), survival stories, changes on efficiency (achieving objectives with minimum resources), changes in equity (fairness in resource and service distribution), performance changes, motivation changes

e. What did people think about the changes you were implementing?

Explore community (women and men), community health services providers, other health providers, health systems level. Probe for any challenges or resistance to the intervention

7. Suggestions

a. What would you change about the HaQIQA intervention?

2. In-depth Interview Guide: Community Health Volunteer Level (English)

Baseline and End line Evaluation

1. Work Background

a. Please tell us about your daily tasks and activities

Probe: Whether they have dual roles and how they manage this e.g. working for different programs and organizations, facility vs. community work. Ask how they manage between volunteer work and their other occupations

b. What do you enjoy about your work as CHW?

c. How happy/ unhappy do you feel to do your work as a community health services provider?

Probe financial incentives; non-financial incentives (material e.g. bicycle / mobile phone or intrinsic, e.g. job satisfaction, impact of work on the community)

Probe for equipment and supplies, workload, working environment, communication, equipment and transportation, safety and sexual harassment, career perspective, community, clients, colleagues, other health workers.

d. How long/ how many years do you intend to work as a community health services provider?

Probe how come? How do you feel about your career prospects and skills development? What will influence how long you intend to stay doing this work?

e. What recognition do you receive for your work?

Probe how this influences motivation or job satisfaction.

Probe recognition from the community / family / organization

2 Supervision

a. Please describe how you have been supervised in your work in the last twelve months?

Probe: who (within the Ministry of Health, CHEW, peer CHW, CHCs, etc.) and for each ask how often does it occur; what does it entail e.g. observation, group meetings, one-on-one meetings; what type of feedback they receive

Probe: When was the last time they were supervised and what happened

b. What changes have been there in the past twelve months in how you are supervised?

Probe: How, what, how come – probe for directives given by supervisors, changes at practice level e.g. ask about changes in referrals, ask about changes in community dialogue days.

Probe: How do you feel about these?

c. What do you feel about the supervision you have received in the last twelve months?

Probe: Does it address your problems and how? What skill development occurs at supervision? How is feedback about your work given to you?

d. How has your work been coordinated in the last twelve months between your different supervisors within the program and others from outside?

Probe: is there coordination e.g. between the CHEWs and CHC members? How do they coordinate themselves? What impact does it have on your work (positive/ negative? Does it affect your motivation? How/How come?

Probe: On coordination for referral and community involvement

e. What do you think the supervision given in the last 12 months was meant to achieve?

f. How could supervision be improved to motivate you further?

3 Referral

a. How did you conduct referral from the community to the facility in the past twelve months?

Probe: Was there a follow up process and how? What were the challenges of the process e.g. availability of referral forms, availability of services at facility, community expectations in the referral process

b. Describe briefly the new tasks and new work tools you were given in order to be able to follow up on referrals

Probe: use of referral tracking register, process of following up on referrals

c. How do you think referral could be improved?

4 Community Engagement

a. How did you been interact with the community in your work aside from visiting them at home in the past twelve months?

Probe: frequency of interaction, where, how it was planned, what interaction entailed

b. How did the community you work in participate in your work in the past twelve months?

Probe: Frequency and type of participation, where, if voluntary, who facilitated the participation and how

Probe: Role of Community Health Committees, presence of dialogue days and action days and how they were planned

c. What was your role in enhancing community participation in the past twelve months?

Probe: Challenges faced in carrying out the role?

5 What worked and what didn't?

a. *What impact did the HaQIQA intervention have on supervision, referral and community engagemant*?

Probe: For each separately - Explore barriers and enablers

b. What aspect of the HaQIQA intervention did you find most effective?

Probe: How come? Why not?

c. Did you encounter difficulties in carrying out your new tasks?

Probe: Did you overcome them? How? How might you overcome them?

d. What other steps could you have taken to make HaQIQA effective?

e. What do people think about the HaQIQA intervention?

Explore community (women and men), community health services providers, other health providers, health systems level. Probe for any challenges or resistance to the intervention

6 Explanations

a. Can you suggest the reasons behind any improvements/changes in supervision, referral, and community engagement in the past twelve months in the community health unit you work in?

Probe on areas respondent identified before as evidence for change
Probe on whether there is a link to the HaQIQA intervention or not and ask for evidence of linkage

b. What enabled the changes brought about by HaQIQA to be/ not be consistently implemented as required?

c. Were there any other positive effects of the change implemented for you and your work that were not expected?

d. Were there any negative effects of the change implemented for you and your work that were not expected?

Probe for comments from clients (women and men), survival stories, changes on efficiency (achieving objectives with minimum resources), changes in equity (fairness in resource and service distribution), performance changes, motivation changes

e. What did people think about the changes you are implementing?

Explore community (women and men), community health services providers, other health providers, health systems level. Probe for any challenges or resistance to the intervention

7 Suggestions

a. What would you change about the HaQIQA intervention?

3. In-depth Interview Guide: Community Health Volunteer (Swahili)

Baseline and End line Evaluation

1. Kuhusu Kazi

a. Tafadhali tueleze kuhusu majukumu na shughuli zako za kila siku.

Chunguza: Iwapo wanamajukumu zaidi ya moja na jinsi wanavyoweza kuyakamilisha e.g. kufanyia kazi vituo na shirika tofauti, kazi zinazofanywa kwa kituo cha afya na zile za kwa kijiji. Uliza jinsi anavyofanya kazi yake ya kujitolea na ile nyengine ya kujikimu

b. Ni kitu gani unachokifurahia kuihusu kazi yako kama CHW?

c. Je, unahisi furaha/ kutokuwa na furaha kiasi gani katika kuifanya kazi yako kama mhudumu wa huduma ya afya ya mashinani?

Chunguza motisha za kifedha; motisha zisizo za kifedha (vifaa kama vile baisikeli /simu za rununu au motisha za kiakili, kwa mfano kuridhika na kazi, athari ya kazi kwa jamii)

Chunguza kuhusu vifaa, uzito wa kazi, mazingira anamofanyiwa kazi, mawasiliano, vifaa na usafirishaji, usalama, usalama na unyanyasaji wa kijinsia, mtazamo wa kikazi, wanajamii, wateja, wenzake kazini, wahudumu wa afya wengineo.

d. Je, unakusudia kuifanya kazi ya mhudumu wa huduma ya afya ya mashinani kwa muda gani/ kwa miaka mingapi?

Chunguza kwa nini? Je, unajiskia vipi kuhusu mwelekeo wa kazi yako na uboreshaji wa ujuzi wako? Je, mambo gani yana athiri muda unaokusudia kuifanya kazi hii?

e. Je, unaupata utambuzi na sifa gani kwa kazi yako?

Chunguza jinsi hii inavyoathiri motisha au kuridhika na kazi?

Chunguza utambuzi kutoka kwa wanajamii/ familia/ shirika

2. Usimamizi

a. Tafadhali nieleze jinsi ulisimamiwa kwenye kazi yako katika miezi kumi na mbili iliyopita?

Chunguza: na nani (kutoka kwa Wizara ya Afya, CHEW, CHW walio viongozi wa CHW wengine, CHCs, na wengine) na uliza jinsi kwa kila mmoja wao usimamizi ulihusu nini kwa mfano kuangaliwa na msimamizi wakati anapofanya kazi, mikutano ya kikundi au ya mmoja kwa mmoja' unapata maoni gani kutoka kwa msimamizi

Chunguza: Mara ya mwisho alipopata usimamizi ni nini kilichotendeka

b. Je, kumekuwa na mabadiliko gani kwenye usimamizi wako katika miezi kumi na mbili iliyopita?

Chunguza: Vipi, nini, mbona - chunguza kuhusu miongozo iliyotolewa na wasimamizi, mabadiliko katika utendakazi kwa mfano uliza kuhusu mabadiliko katika uelekezaji, mabadiliko katika majadiliano ya wahudumu wa afya na wanajamii.

Chunguza: Unajihisi vipi kuyahusu haya?

c. Je unayo maoni gani kuhusu usimamizi uliopata katika miezi kumi na mbili iliyopita?

Chunguza: Hukusaidia kutatua shida zako na kivipi? Ni ujuzi upi unaokuzwa wakati wa usimamizi? Unapatiwa aje maoni kuhusu kazi yako?

d. Je, uratibu wa kazi yako umefanywa aje katika miezi kumi na mbili iliyopita kati ya wasimamizi wako tofauti walio kwenya huduma ya afya jamiI na wale wengine?

Chunguza: Je, kunao uratibu kwa mfano kati ya CHEW na memba wa CHC? Hao hujiratibu vipi? Je, hii ina athari gani kwa kazi yako(nzuri/ mbaya)? Je, hili inaiathiri motisha yako? Vipi/ Mbona?

Chunguza: Kuhusu uratibu wa uelekezaji na kuhusisha wanajamii

e. Unaonelea usimamizi uliotolewa katika miezi 12 iliyopita ulikusudia kuyafikia malengo gani?

f. Usimamizi unaeza kuboreshwa vipi ili kukutia motisha zaida?

3. Uelekezi

a. Je, ulifanya aje uelekezi kutoka kwa jamii hadi kituo cha afya katika miezi kumi na mbili iliyopita?
Chunguza: Kuna mpango ulikuwepo wa kufuatilia uelekezi na ulikuwa upi? Ni matatizo gani yaliyokumba uelekezi kwa mfano uwepo wa fomu za uelekezi, uepo wa huduma katika kituo cha afya, matarajio ya jamii katika taratibu ya uelekezi?

b. Eleza kwa ufupi majukumu na vifaa vipya vya kazi ulivyopewa ili kukuwezesha kufuatilia uelekezi
Chunguza: kwa rejesta ya kufuatilia walioelekezwa, taratibu ya kufuatilia uelekezi

c. Unafikiria uelekezi unaweza kuboreshwa vipi?

4. Kuhusiha jamii

a. Ulikutana wapi na wanajamii katika kazi yako kando na kuwatembelea nyumbani katika miezi kumi na mbili iliyopita?
Chunguza: Mikutano ilifanyika mara ngapi, wapi, ilipangwa vipi, mikutano ilihusu nini?

b. Jamii/ Kijiji unamofanyai kazi ilishiriki vipi katika kazi yako katika miezi kumi na mbili iliyopita?
Chunguza: Kiwango cha na aina ya kujishirikisha, wapi, iwapo ilikuwa ya kujitolea, nani alipanga kushiriki kwao.
Chunguza: Jukumu la CHC (kamati ya afya ya jamii), uwepo wa siku za majadiliano (dialogue days) na jinsi zilivyopangwa

c. Jukumu lako katika kuhusisha wanajamii umekuwa upi katika miezi kumi na mbili iliyopita?
Chunguza: Matatizo yaliyokukumba katika jukumu hili?

5. Kilichofanikiwa na kilichokosa kufanikiwa

a. Je, mradi wa HaQIQA ulikuwa na athari gani katika usimamizi, uelekezi na kuhusishwa kwa jamii?

Chunguza: kwa kila mmoja kando - Vichunguze vikwazo na viwezeshaji

b. Je, ni mambo gani ya mradi wa HaQIQA uliyoyaona yakifanya kazi vizuri zaidi?

Chunguza: Mbona hivyo/ sivyo?

c. Je, ulikumbana na matatizo katika kuyatekeleza majukumu yako mapya?

Chunguza: Je, uliweza kukabiliana nazo? Kivipi? Je, unawezaje kukabiliana nazo?

d. Je, ungezichukua hatua zipi zinginezo ili kuufanya HaQIQA kuwa wa kufana zaidi?

e. Je, watu wanachukulia vipi mradi wa HaQIQA?

Chunguza kuhusu wanajamii (wanawake na wanaume), wahudumu wa huduma ya afya ya mashinani, wahudumu wa afya wengine, walio kwenye kiwango cha mfumo wa afya. Chunguza kuhusu matatizo au upinzani wowote dhidi ya mradi

6. Maelezo

a. Je, unaweza kutoa sababu kuhusu uboreshaji/ mabadiliko yoyote katika usimamizi, uelekezi, na kuhusishwa kwa jamii katika miezi kumi na mbili iliyopita kwenye unit unamofanyia kazi?

Chunguza sehemu zilzotajwa na mhojiwa hapo awali kama ushahidi wa mabadiliko
Chunguza iwapo kuna uhusiano na mradi wa HaQIQA au la na uliza kuhusu ushahidi wa uhusiano

b. Ni nini kilichosababisha utekelezaji/ kutotekelezwa kwa mabadiliko yaliyoletwa na HaQIQA kwa njia inayotakikana?

c. Je, kunazo athari zozote nzuri zilizoletwa na mabadiliko yaliyofanywa kwako na kwa kazi yako, ambazo hazikuwa zimetarajiwa?

d. Je, kunazo athari zozote mbaya zilizoletwa na mabadiliko yaliyofanywa kwako na kwa kazi yako, ambazo hazikuwa zimetarajiwa?

Chunguza maoni kutoka kwa wateja (wanawake na wanaume), hadithi za walioponea, mabadiliko ya ufanisi, mabadiliko kwa usawa na haki katika kupata huduma kwa wanajamii, mabadiliko ya utendakazi, mabadiliko kwa motisha

e. Je, watu wanachukulia vipi mabadiliko unayotekeleza?

Chunguza kuhusu wanajamii (wanawake na wanaume), wahudumu wa huduma ya afya ya mashinani, wahudumu wa afya wengine, walio kwenye kiwango cha mfumo wa afya. Chunguza kuhusu matatizo au upinzani wowote dhidi ya mradi

7. Mapendekezo

a. Je, ungebadilisha nini kuhusu mradi wa HaQIQA?

4. Supervisor In-depth Interview Guide: Supervisor Level (Non CHEWs) - English

Baseline and End Line Evaluation

1. Work Background

f. Please tell us about your tasks and activities as a supervisor in the community health strategy

Probe: Whether they have dual roles and how they work around this e.g. working for different programs and organizations, facility vs. community work.
If CHW or CHC member ask how they juggle between volunteer work and their other occupations

g. What do you enjoy about your work as a supervisor?

2. Supervision

NB: 2a to 2c ask for peer CHWs only, for other supervisors skip to 2d

a. What changes were there in the past twelve months in how you are supervised?

Probe: How, what, how come – probe for directives given by supervisors, changes at practice level

Probe: How do you feel about these?

b. How have you been supervised in your work in the last twelve months?

Probe: who (within the Ministry of Health, CHEW, peer CHW, CHCs, etc.) and for each ask for how and frequency, whether supervisors offered training, how feedback is given etc.

h. Did you have regular meetings with your supervisor(s) in the last twelve months and what did these meetings entail?

Probe: ask for frequency of meetings; type of meetings e.g. one-on-one, group, observation; who facilitated e.g. CHEW, CHCs, peer CHWs, etc; agenda of meetings

ALL SUPERVISORS

i. Kindly describe how you have supervised CHWs/CHEWs in the past twelve months?

Probe: With who, How, what, how come – probe for directives given to CHWs/ CHEWs, changes at practice level e.g. ask about how they supervised e.g. meetings, one-on-one; ask about frequency of supervision; observed parctice

Probe: How do you feel about these?

j. How frequently did you meet with CHWs/ CHEWs in the last twelve months and what did these meetings entail?

Probe: ask for frequency of meetings; type of meetings e.g. one-on-one, group, observation; who facilitated e.g. CHEW, CHCs, peer CHWs, etc; agenda of meetings

k. Describe briefly the new tasks and/ or new work tools you were given in order to be able to carry out effective supervision

Probe: barriers and effectiveness of use of supervision checklists and forms, carrying out group supervision, conducting supportive supervision, execution of problem solving and advocacy plans

l. What do you think the supervision you gave in the last 12 months was meant to achieve? *Unaonelea usimamizi uliotowa katika miezi 12 iliyopita ulikusudia kuyafikia malengo gani?*

3. Referral

a. How did referral occur from the community to the facility in the past twelve months?

Probe: Was there a follow up process and how? What were the challenges of the process e.g. availability of referral forms, availability of services at facility, community expectations in the referral process

b. Describe briefly the new tasks and new work tools that the CHWs were given in order to be able to follow up on referrals

Probe: use of referral tracking register, process of following up on referrals

c. How do you think referral could be improved?

4. Community Engagement

d. How have you been interacting with the community in your work aside from supervising CHWs/ CHEWs in the past twelve months?

Probe: frequency of interaction, where, how it was planned, what interaction entailed

e. How has the community you work in been participating in your supervision work in the past twelve months?

Probe: Frequency and type of participation, where, if voluntary, who facilitated the participation and how

Probe: Role of Community Health Committees, presence of dialogue days and action days and how they were planned

f. What has been your role in enhancing community participation in the past twelve months?

Probe: Challenges faced in carrying out the role?

5. What worked and what didn't?

e. What impact did the HaQIQA intervention have on supervision, referral and community engagemant?

Probe: For each separately - Explore barriers and enablers

f. What aspect of the HaQIQA intervention did you find most effective?

Probe: How come? Why not?

g. Did you encounter difficulties in carrying out your new tasks?

Probe: Did you overcome them? How? How might you overcome them?

h. What other steps could you have taken to make HaQIQA effective?

6. Explanations

f. Can you suggest the reasons behind any improvements/changes in supervision, referral, and community engagement in the past twelve months in the community health unit you work in?

Probe on areas respondent identified before as evidence for change
Probe on whether there is a link to the HaQIQA intervention or not and ask for evidence of linkage

g. What enabled the changes brought about by HaQIQA to be/ not be consistently implemented as required?

h. Were there any other positive effects of the change implemented for you and your work that were not expected?

i. Were there any negative effects of the change implemented for you and your work that were not expected?

Probe for comments from clients (women and men), survival stories, changes on efficiency (achieving objectives with minimum resources), changes in equity (fairness in resource and service distribution), performance changes, motivation changes

j. What did people think about the changes you were implementing?

Explore community (women and men), community health services providers, other health providers, health systems level. Probe for any challenges or resistance to the intervention

7. Suggestions

b. What would you change about the HaQIQA intervention?

5. Supervisor In-depth Interview Guide: Supervisor Level (Non CHEWs) - Swahili

Baseline and End Line Evaluation

1. Kuhusu Kazi

a. Tafadhali tueleze kuhusu majukumu na shughuli zako kama msimamizi katika mradi wa serikali wa huduma ya afya ya jamii.

Chunguza: Iwapo wanamajukumu zaidi ya moja na jinsi wanavyoweza kuyakamilisha e.g. kufanyia kazi vituo na shirika tofauti, kazi zinazofanywa kwa kituo cha afya na zile za kwa kijiji.

Iwapo ni CHW au memba wa CHC uliza jinsi anavyafanya kazi yake ya kujitolea na ile nyengine ya kujikimu

b. Ni kitu gani unachokifurahia kuihusu kazi yako kama msimamizi?

2. Usimamizi

KUMBUKA: 2a na 2c waulize CHW walio viongozi wa timu na CHEW pekee, kwa wasimamizi wengine ruka hadi 2d

a. Je, kumekuwa na mabadiliko gani kwenye usimamizi wako katika miezi kumi na mbili iliyopita?

Chunguza: Vipi, nini, mbona - chunguza kuhusu miongozo iliyotolewa na wasimamizi, mabadiliko katika utendakazi

Chunguza: Unajihisi vipi kuyahusu haya?

b. Umesimamiwa aje kwa kazi yako katika miezi kumi na mbili iliyopita?

Chunguza: na nani (kutoka kwa Wizara ya Afya, CHEW, CHW walio viongozi wa CHW wengine, CHCs, na wengine) na uliza jinsi kila mmoja wao anavyosimamia na mara ngapi, iwapo wasimamizi huwapatia mafunzo, jinsi wasimamizi wanavyotoa maoni, na mengineyo

c. Ulikuwa na mikutano za mara kwa mara na wasimamizi wako katika miezi sits iliyopita na na mikutano haya yalihusu nini?

Chunguza: uliza mikutano hufanyika mara ngapi; aina ya mikutano kwa mfano mmoja kwa mmoja, ya kikundi, ya kutazamwa unavyofanya kazi; ni msimamizi yupi aliyeongoza mkutano kwa mfano CHEW, CHC, CHW walio viongozi wa CHW wengine, na wengineo; lengo za mikutano

KWA WASIMAMIZI WOTE

d. Tafadhali eleza jinsi ulivyosimamia CHW/ CHEW katika miezi kumi na mbili iliyopita?

Chunguza: Na nani, Vipi, nini, mbona - chunguza kuhusu miongozo iliyotolewa kwa CHW/ CHEW, mabadiliko katika utendakazi kwa mfano uliza jinsi walivyotoa usimamizi kwa mfano kupitia mikutano, kutana mmoja mmoja; uliza ilifanyika mara ngapi, iwapo aliweza kuwatazama walipotekeleza majukumu yao.

Chunguza: Unajihisi vipi kuyahusu haya?

e. Ulikuwa na mikutano za mara kwa mara na CHW/ CHEW katika miezi sita iliyopita na mikutano haya yalihusu nini?

Chunguza: uliza mikutano hufanyika mara ngapi; aina ya mikutano kwa mfano mmoja kwa mmoja, ya kikundi, ya kutazamwa unavyofanya kazi; ni msimamizi yupi aliyeongoza mkutano kwa mfano CHEW, CHC, CHW walio viongozi wa CHW wengine, na wengineo; lengo za mikutano

f. Eleza kwa ufupi majukumu na/ au vifaa vipya vya kazi ulivyopewa ili kukuwezesha kutekeleza majukumu yako barabara kama msimamizi

Chunguza: yanayotatiza na uwezekano wa kutumia ratiba na fomu za usimamizi, kutekeleza usimamizi katika mkutano wa kikundi, kutekeleza usimamizi unaojali masilahi ya wafanyi kazi, kutekekelza mipango ya kutatua shida na ya utetezi wa masilahi

g. Unaonelea usimamizi uliotowa katika miezi 12 iliyopita ulikusudia kuyafikia malengo gani?

3. Uelekezi

a. Je, uelekezi ulifanywa aje kutoka kwa jamii hadi kituo cha afya katika miezi kumi na mbili iliyopita?

Chunguza: Kuna mpango ulikuwepo wa kufuatilia uelekezi na ulikuwa upi? Ni matatizo gani yaliyokumba uelekezi kwa mfano uwepo wa fomu za uelekezi, uepo wa huduma katika kituo cha afya, matarajio ya jamii katika taratibu ya uelekezi?

b. Eleza kwa ufupi majukumu na vifaa vipya vya kazi vilivyopewa CHW ili kuwawezesha kufuatilia uelekezi

Chunguza: kwa rejesta ya kufuatilia walioelekezwa, taratibu ya kufuatilia uelekezi

c. Unafikiria uelekezi unaweza kuboreshwa vipi?

4. Kuhusisha jamii

a. Umekutana wapi na wanajamii katika kazi yako kando na kuwasimamia CHW/ CHEW katika miezi kumi na mbili iliyopita

Chunguza: Mikutano ilifanyika mara ngapi, wapi, ilipangwa vipi, mikutano ilihusu nini?

b. Jamii/ Kijiji unamofanyia kazi imeshiriki vipi katika kazi yako kama msimaizi katika miezi kumi na mbili iliyopita?

Chunguza: Kiwango cha na aina ya kujishirikisha, wapi, iwapo ilikuwa ya kujitolea, nani alipanga kushiriki kwao.

Chunguza: Jukumu la CHC (kamati ya afya ya jamii), uwepo wa siku za majadiliano (dialogue days) na jinsi zilivyopangwa

c. Jukumu lako katika kuhusisha wanajamii umekuwa upi katika miezi kumi na mbili iliyopita?
Chunguza: Matatizo yanayokukumba katika jukumu hili?

5. Kilichofanikiwa na kilichokosa kufanikiwa

a. Je, mradi wa HaQIQA ulikuwa na athari gani katika usimamizi, uelekezi na kuhusishwa kwa jamii?
Chunguza: kwa kila mmoja kando - Vichunguze vikwazo na viwezeshaji

b. Je, ni mambo gani ya mradi wa HaQIQA uliyoyaona yakifanya kazi vizuri zaidi?
Chunguza: Mbona hivyo/ sivyo?

c. Je, ulikumbana na matatizo katika kuyatekeleza majukumu yako mapya?
Chunguza: Je, uliweza kukabiliana nazo? Kivipi? Je, unawezaje kukabiliana nazo?

d. Je, ungezichukua hatua zipi zinginezo ili kuufanya HaQIQA kuwa wa kufana zaidi?

6. Maelezo

a. Je, unaweza kutoa sababu kuhusu uboreshaji/ mabadiliko yoyote katika usimamizi, uelekezi, na kuhusishwa kwa jamii katika miezi kumi na mbili iliyopita kwenye unit unamofanyia kazi?
Chunguza sehemu zilzotajwa na mhojiwa hapo awali kama ushahidi wa mabadiliko
Chunguza iwapo kuna uhusiano na mradi wa HaQIQA au la na uliza kuhusu ushahidi wa uhusiano

b. Ni nini kilichosababisha utekelezaji/ kutotekelezwa kwa mabadiliko yaliyoletwa na HaQIQA kwa njia inayotakikana?

c. Je, kunazo athari zozote nzuri zilizoletwa na mabadiliko yaliyofanywa kwako na kwa kazi yako, ambazo hazikuwa zimetarajiwa?

d. Je, kunazo athari zozote mbaya zilizoletwa na mabadiliko yaliyofanywa kwako na kwa kazi yako, ambazo hazikuwa zimetarajiwa?
Chunguza maoni kutoka kwa wateja (wanawake na wanaume), hadithi za walioponea, mabadiliko ya ufanisi, mabadiliko kwa usawa na haki katika kupata huduma kwa wanajamii, mabadiliko ya utendakazi, mabadiliko kwa motisha

e. Je, watu walichukulia vipi mabadiliko uliyotekeleza?
Chunguza kuhusu wanajamii (wanawake na wanaume), wahudumu wa huduma ya afya ya mashinani, wahudumu wa afya wengine, walio kwenye kiwango cha mfumo wa afya. Chunguza kuhusu matatizo au upinzani wowote dhidi ya mradi

7. Mapendekezo

a. Je, ungebadilisha nini kuhusu mradi wa HaQIQA?

6. Supervision tracking questionnaire

Community Unit(s) / Research Site(s) ………………………………. ………………………………. Data collection Month …………………………………

Interviewer………………………………………………………………………………………………. Respondent Designation…………………………………………………………………

ONE-ON-ONE SUPERVISION MEETING WITH UNIT CHWs (including lead CHWs)<br>*Ask all respondents*<br>*(Record each meeting per row)*<br>Total no. of<br>meetings=	Date	# and Designation of Supervisors in the meeting e.g. 1CHC, 1DCHS		Demographics of meeting convener				# and Designation of Supervisee participants e.g. 1 CHW	Meeting recorded in One-on-One Supervision Tool Y/N	Recorded elsewhere Y/N<br>If Y, specify	Verification of recording in One-on-One Supervision Tool by Researcher<br>Y/N	Topics tackled at meeting and additional comments (include comments on use of One-on-one Supervision Tool)	
		Male	Female	Designation e.g. CHEW, SCASCO	Sex	Profession e.g. nurse	Years of service in CHS	Male	Female				

													Nxt . Pg
													Nxt . Pg
													Nxt . Pg

1. SUPERVISION AND COORDINATION ACTIVITIES

Key

Nxt. Pg – For this section write on next page

Instructions: Topics tackled at meeting and additional comments (include comments on use of One-on-one Supervision Tool)

**Meeting Date**	**Topic Tackled**	**General Comments**	**Comments on One-on-one Supervision Tool**

GROUP SUPERVISION of CHWs (include those done by and for lead CHWs)<br>*Ask all respondents*	Date	# and Designation of Supervisors in the meeting e.g. 1CHC, 1Peer CHW		Demographics of supervisor who convened the meeting				# and Designation of Supervisee participants e.g. 6 CHWs		Meeting recorded in Group Supervision Tool Y/N	Minutes Recorded Y/N	Verification of recording in Group Supervision Tool by Researcher<br>Y/N	Topics tackled at meeting and additional comments (include comments on use of Group Supervision Tool)
		Male	Female	Designation e.g. CHEW, SCASCO	Sex	Profession	Years of service in CHS	Male	Female				
													Nxt. Pg
													Nxt. Pg
													Nxt. Pg
													Nxt. Pg
													Nxt. Pg
(Record each meeting per row)													Nxt. Pg
Total no. of meetings=													Nxt. Pg
													Nxt. Pg
													Nxt. Pg

Key

Nxt. Pg – For this section write on next page

Instructions: Topics tackled at meeting and additional comments (include comments on use of Group Supervision Tool)

Meeting Date	Topic Tackled	General Comments	Comments on Group Supervision Tool

HOME VISIT OBSERVATION made to CHWs *Ask all respondents*	Date	# and Designation of Supervisors carrying out assessment e.g. 1CHC, 1CHEW		Demographics of supervisor who planned and facilitated the observation				# and Designation of Supervisees assessed e.g. 1 CHW		Observation recorded in Home Visit Observation Tool Y/N	Recorded elsewhere Y/N<br>If Y, specify	Verification of recording in Home Visit Observation Tool by Researcher<br>Y/N	Issues observed by supervisor(s) at visit and additional comments (include comments on use of Home Visit Checklist Tool)
		Male	Female	Designation e.g. CHEW, SCASCO	Sex	Profession	Years of service in CHS	Male	Female				
													Nxt. Pg
													Nxt. Pg

(Record each day of observation per row)<br>Total days of observations													Nxt. Pg

Key

Nxt. Pg – For this section write on next page

Instructions: Issues observed by supervisor(s) at visit and additional comments (include comments on use of Home Visit Checklist Tool)

**Observation Date**	**Issues Observed**	**General Comments**	**Comments on Home Visit Checklist Tool**

SPOT CHECKS conducted for CHWs assignment areas<br>*Ask all respondents*	Date	# and Designation of Supervisors carrying out assessment e.g. 1CHC, 1CHEW		Demographics of supervisor who planned and facilitated the spot checks				# and Designation of Supervisees working in the assessed area(s) e.g. 2 CHWs		Assessment recorded in Spot Checks Tool<br>Y/N	Recorded elsewhere Y/N<br>If Y, specify	Verification of recording in Spot Checks Tool by Researcher<br>Y/N	Issues observed by supervisor(s) at spot checks and additional comments (include comments on use of Spot Checks Tool)
		Male	Female	Designation e.g. CHEW, SCASCO	Sex	Profession	Years of service in CHS	Male	Female				
(Record each day of spot check per row)<br>Total days of spot check=													Nxt. Pg
													Nxt. Pg
													Nxt. Pg

Key

Nxt. Pg – For this section write on next page

Instructions: Issues observed by supervisor(s) at spot checks and additional comments (include comments on use of Spot Checks Tool)

Spot Check Date	Issues Observed	General Comments	Comments on Spot Checks Tool

ONE-ON-ONE SUPERVISION MEETING FOR THE CHEW<br>*Ask CHEW and their supervisors only*	Date	# and Designation of Supervisors in the meeting e.g. 1Facility In-Charge, 1DCHS		Demographics of supervisor who facilitated the one-on-one supervision				# and Designation of Supervisee participants e.g. 1 CHEW		Meeting Recorded Y/N<br>If Y, specify where	Topics tackled at meeting and additional comments
		Male	Female	Designation e.g. CHEW, SCASCO	Sex	Profession	Years of service in CHS	Male	Female		
											Nxt. Pg
											Nxt. Pg
											Nxt. Pg
(Record each meeting per row)<br>Total meetings=											Nxt. Pg

Key

Nxt. Pg – For this section write on next page

Instructions: Topics tackled at meeting and additional comments

Meeting Date	Topics Tackled	General Comments
